# Personalized Machine Learning Intervention to Improve Sleep Quality Using Wearable Technology in Healthy Middle-Aged Adults From Mexico City: Protocol for a Pilot Randomized Controlled Trial

**DOI:** 10.2196/76415

**Published:** 2026-01-06

**Authors:** Rodrigo Quezada Reyes, Luis A Trejo

**Affiliations:** 1 Computer Science Department, School of Engineering and Science, Tecnologico de Monterrey, Carretera al Lago de Guadalupe Km 3.5, Col. Margarita Maza de Juarez, Atizapán de Zaragoza, Estado de México, 52926, Mexico, 52 5558645555

**Keywords:** sleep quality, well-being, machine learning, smartwatches, sensors, Pittsburgh Sleep Quality Index, PSQI, wearables

## Abstract

**Background:**

In 2019, global sleep surveys reported that 80% of adults want to improve their sleep quality, and in 2021, 45% were reported to be dissatisfied with their sleep. In 2025, among American adults, 37% reported sleep dissatisfaction and 38% reported not feeling energized after sleep. These findings are consistent with data from the 2016 nationally representative survey of Mexican adults (aged ≥18 years), in which 37% reported sleep problems.

**Objective:**

This protocol describes a pilot randomized controlled trial (RCT) testing whether a single personalized sleep intervention driven by machine learning (ML) using consumer wearable data can improve sleep scores compared with generic sleep hygiene education in healthy middle-aged adults from Mexico City.

**Methods:**

This pilot RCT plans to enroll 32 participants (16 per arm; stratified by sex) in Mexico City. All participants wear Samsung Galaxy Watch 4 devices for 60 days. During days 1‐30 (baseline), objective sleep data (10 variables related to duration, efficiency, sleep stages, movements, cycles, and recovery metrics) are collected. The control group receives generic sleep hygiene education. The experimental group receives personalized recommendations on day 30 based on the top predictive sleep parameters identified by ML models using Shapley Additive Explanations analysis and recursive feature elimination. The primary outcome is the sleep score (scale 1‐100; composite device metric) during days 31‐60, which is analyzed using analysis of covariance, with the baseline sleep score as a single covariate. The secondary outcome is the Pittsburgh Sleep Quality Index (PSQI) global score (scale 0‐21; subjective validation), which is assessed at baseline, day 30, and day 60.

**Results:**

The study is currently in progress. Recruitment started in August 2024 and ended in July 2025. Data collection is expected to be completed by December 2025. The study will compare 960 nights from the control group with 960 nights from the experimental group to explore whether ML interventions can improve sleep scores using wearable technology, generate a dataset from objective data for iterative model training and analysis, correlate objective and subjective sleep quality metrics, and establish whether a feasible framework for proactive sleep quality approaches can be developed. The results will be available by March 2026, and the findings will be submitted for publication within 6 months of study completion.

**Conclusions:**

This pilot study establishes the feasibility and preliminary effect size for ML-personalized sleep interventions using consumer wearables within a manufacturer-independent personalization framework. The approach combines objective device monitoring with a subjective measure (PSQI) to test whether precision targeting of individual sleep parameters outperforms generic recommendations. If validated, this methodology could advance sleep interventions from universal protocols toward individualized behavioral targeting. The resulting dataset will enable model refinement and provide preliminary evidence for scaling personalized sleep health interventions in healthy populations.

## Introduction

### Overview

Sleep is essential for health and productivity. Global sleep dissatisfaction has been reported by 35%‐45% of adults, and this is consistent with 37% of Mexican adults reporting sleep problems [1-5]. Sleep benefits are well researched, and they include (1) a healthy body through immune system efficiency [6,7], (2) healthy emotions through psychological well-being [8], and (3) a healthy mind through memory consolidation [9-11]. The negative consequences of poor sleep are also well researched, and they include heart disease [12], metabolic syndrome [13], high blood pressure [14], diabetes [15], obesity [16], common cold [6], higher probability of injury [17], and higher mortality risk [18-20].

### Demographic and Geographic Context

This pilot study will be conducted in Mexico City, a metropolitan area with over 21 million residents [21]. Adults aged 30‐60 years represent over 40% of Mexico’s economically active population [22], making this demographic relevant for sleep health research. Urban workers in Mexico City face unique sleep challenges, including long commutes, shift work, and environmental stressors, that may impact sleep quality [5,23,24]. National surveys indicate that 35%‐40% of Mexican adults sleep fewer than 7 hours per night, with 37% reporting sleep problems [4,5]. Mexico City’s context provides a useful setting to explore sleep intervention feasibility in a rapidly urbanizing environment [25,26].

### Knowledge Gap

Despite growing interest in wearable sleep technology, a significant knowledge gap exists regarding whether machine learning (ML) analysis of individual sleep patterns from commercial devices can generate actionable recommendations that improve objective sleep outcomes. No previous studies have examined whether sleep scores from wearables (Samsung Galaxy Watch [27] or equivalent) can serve as primary outcome measures for personalized ML-derived interventions validated against the Pittsburgh Sleep Quality Index (PSQI) [28]. Existing consumer sleep coaching, such as Samsung’s Sleep Coach, categorizes users into predefined “sleep animal” types based on general sleep patterns (sleep time, sleep consistency, and awake time) and provides standardized multiweek programs [29,30] without subjective validation. Recent advances focus on validation studies comparing device accuracy to polysomnography (PSG) and developing ML algorithms for sleep stage classification [31-34]. Samsung has collected over 716 million nights of sleep data (collected between June 2021 and May 2023 via the Galaxy Watch series) globally [35], and research using Samsung Galaxy Watch sleep scores as intervention targets remains unexplored.

### Study Uniqueness

This pilot study addresses the gaps in the literature by exploring if wearable sleep scores [27,29,30,36-43] can serve as primary outcome measures for personalized ML-derived interventions and showing if ML analysis of individual patterns can identify the predictors of improvement, with validation against the PSQI (structured 30-day correlation framework) [28], in healthy middle-aged Mexican adults, a population facing unique urban sleep challenges but underrepresented in sleep technology research. This represents a personalized, ML-driven sleep intervention using a consumer wearable sleep data source (sleep metrics) for personalization as well as primary outcome measures (sleep scores) and user subjective assessments, establishing a novel paradigm for sleep health.

### Core Methodological Innovation

This pilot study tests a single, focused innovation: a personalization framework that generates behavioral sleep interventions from ML analysis of individual patterns in consumer wearable data. Our innovation is the *methodology*, not the device, theories, or algorithms. We are not validating device accuracy or comparing algorithms. Existing studies have established these foundations [36-43]. We are testing whether personalized behavioral interventions derived from individual ML analysis of the sleep metrics of a consumer device can improve objective sleep scores better than generic sleep hygiene education. This methodology is intentionally device-agnostic. The same personalization framework can be applied to other manufacturers’ wearables, providing continuous numeric sleep data and sleep score scales. We perform tests within Samsung’s ecosystem to isolate the intervention effect (personalized vs generic) without confounding by device differences. If successful, this will establish a transferable approach that other researchers can replicate across manufacturers, potentially driving the field toward less proprietary and more user-centered sleep optimization tools.

### Sleep Measurements

There are 2 ways to measure sleep. The first involves subjective measures of sleep that assess patient-reported satisfaction and functional impact (essential domains reflecting clinical relevance) and have evolved from sleep diaries [44] into validated survey instruments like the gold-standard PSQI [28] for sleep quality and sleep patterns, the Epworth Sleepiness Scale for daytime sleepiness [45], the Patient-Reported Outcomes Measurement Information System for sleep disturbances [46], and the Sleep Health Index for general sleep health [47]. The second involves objective measures of sleep that capture physiological parameters not consciously perceived (sleep parameters [measurable components] such as efficiency, latency, architecture, and fragmentation). Sensors collect objective measures of sleep by measuring physical inputs [31-34,48-51]. The most important biomarkers for sleep tracking are heart rate, breathing rate, blood oxygen flow, and body movements [51,52].

Sleep devices can be subdivided by grade into (1) medical-grade devices (also called clinical-grade devices), such as the gold-standard PSG [53,54]; and (2) commercial-grade devices (also called consumer-grade devices or consumer sleep technology [55]), such as off-the-shelf smartwatches [43]. They can also be subdivided by their body proximity into (1) devices in contact with the body (wearables) like watches, patches, rings, and bands [56]; (2) devices near the body (nearables) like bedside stands and devices in or under the mattress [57]; and (3) devices inside the body (implants) like subcutaneous electroencephalography devices [58,59]. Crivello et al [60] categorized sleep monitoring technologies by deployment as follows: laboratory-based (eg, PSG and specialized electroencephalography), ambulatory or portable (eg, wearables, on-bed sensors, actigraphy devices, and contactless devices), and consumer integration (eg, smartphone apps and smart home sensors).

In 2019, the wearable sleep tracker segment represented 44% of global smart sleep-tracking products [57]. The most common body locations of wearables are the head, chest, wrist, and finger. The wrist is the most popular location, representing more than 58% of the segment in 2024 [61].

PSG is the gold standard for objective sleep measures, but requires expensive equipment. Type 1 PSG requires technologist labor and lab settings, while type 2 PSG can be performed at home with or without a technologist [53,54,62-66]. The cost of long-term sleep monitoring with type 1 PSG or type 2 PSG is very high [66-68]. However, PSG is instrumental in setting a baseline to compare recent technological advances with wearable objective measurements for identifying an affordable and noninvasive option for continuous monitoring [36,62,69-71].

As the lines between consumer and clinical devices are blurring [55], there is an ongoing debate regarding the trustworthiness of available technologies. In 2018, the American Academy of Sleep Medicine stated that for consumer sleep technologies to provide a diagnosis or treatment, they first need Food and Drug Administration clearance and testing against current gold standards [72]. Studies have reported overall overestimated sleep with these devices compared with PSG due to lower sensitivity to detect wake epochs [73] and more discrepancies in sleep disorder populations [74-76].

Commercial-grade wearable devices should be validated with PSG to ensure reliability. A study in 2017 reported high agreement between a commercial wearable device and PSG in good sleepers but low agreement in patients with insomnia [75]. Another study in 2019 showed comparable results for the rapid eye movement (REM) sleep phase [63]. A study in 2020 concluded that wearables can accurately measure sleep and cardiorespiratory variables in healthy people [77], and a recent study reported the accuracy of a wearable device in sleep stage identification [65].

This study focuses on healthy adults without diagnosed sleep disorders [78-81], as this population represents 63%‐67% of adults [82,83]. In 2004, only 9%-11% of people were considered to have chronic insomnia by the World Health Organization Regional Office for Europe [84]. In 2022, a study reported that 10%-15% of people have chronic insomnia with daytime consequences [85], and another source reported that up to 22% of people meet the insomnia sleep disorder criteria [86]. These findings align with the overall percentage of adults not getting enough sleep (33.3%-36.8%) from 2013 to 2022 [82,83].

### Sleep Quantity

Sleep quantity refers to the amount of daily time spent sleeping (sleep duration), measured in hour units. The American Academy of Sleep Medicine, the Sleep Research Society, and the National Sleep Foundation agree that adults (aged 18‐60 years) need a minimum of 7 hours of sleep each night [87,88], and a sleep duration less than that implies a sleep deprivation condition (insufficient sleep) [18]. The following variables can be considered for assessment: (1) time in bed (TIB), period from lights off to lights on; (2) total sleep time (TST), actual time spent sleeping (sleep quantity) consistently shorter than TIB unless efficiency reaches 100%; and (3) sleep efficiency ([TST / TIB] × 100).

There are four main disadvantages of relying on this metric in isolation: (1) quantity increase does not guarantee a positive correlation with sleep quality (eg, during the 2021 pandemic period, Americans were sleeping more [quantity], but sleep quality declined) [89]; (2) TIB can be misunderstood as TST (these are only equal if 100% sleep efficiency is achieved) [90-92]; (3) sleeping beyond a limit can have negative consequences, such as stroke [93] and intracerebral hemorrhage [94] (the National Sleep Foundation has recommended a maximum sleep duration of 9 hours [88]); and (4) sleeping time cannot provide valuable data on sleep architecture, including time spent in each of the 4 sleep stages, number of sleep cycles, distribution of sleep stages, and transition speed between sleep phases [90-92].

### Sleep Quality: Definition and Measurement

Sleep quality or restorative sleep represents a multidimensional construct encompassing both objective parameters and subjective restoration. Nelson et al [95] identified 4 core attributes: sleep efficiency, latency, duration, and wake after sleep onset. Contemporary understanding extends beyond these continuity attributes to include architecture components (adequate deep sleep for physical recovery, sufficient REM sleep for cognitive restoration, and optimal 4‐6 sleep cycles) and minimal fragmentation [95-99]. An increase in quantity does not guarantee a positive correlation with sleep quality [89]. The 2014 and 2015 survey results by the National Sleep Foundation showed an 11-point delta in sleep duration compared with the sleep quality score and a final 69/100 score for sleep quality [47]. Despite China’s records showing that the general population has an average sleep duration of 7.31 hours [100] and Chinese university students reporting an average sleep duration of 7.08 hours [101], studies on the prevalence of poor sleep quality have been published [102].

### Sleep Quality in Healthy Populations

Rosipal et al [103] identified objective components for sleep quality indexing in normal sleep. Moreover, O'Donnell et al [104] found significant subjective-objective relationships in healthy older study participants. Even healthy individuals show consequences, including cognitive decrements, mood alterations, fatigue, and behavioral adaptations (increased caffeine use).

### Related Studies

In 2018, Guillodo et al [105] concluded that wearables for sleep monitoring are acceptable, and de Arriba-Pérez et al [71] concluded that the use of consumer-grade wearables as sleep indicators is a feasible option, although some require more rigor. In 2023, Lins et al [106] concluded that the photoplethysmography technology of the Samsung Galaxy Watch 4 was comparable to the oscillometric reference method for blood pressure determination. Moreover, Gaiduk et al [36] reported the feasibility of measuring several relevant sleep characteristics with Samsung Galaxy Watch 4 as a sensor-based approach in a real-life setting, if some level of inaccuracy is accepted. In 2024, Alshamari and Althobaiti [107] compared wearable sleep tracker–related improvements and concluded that a WHOOP device can both improve the perception of quality and provide accurate measures of sleep and cardiorespiratory variables.

## Methods

### Conformance to Reporting Standards

This protocol conforms to the SPIRIT (Standard Protocol Items: Recommendations for Interventional Trials) 2013 Statement. The SPIRIT 2025 checklist of items to address in a randomized trial protocol is presented in Checklist 1.

### Trial Design

This is a parallel-group randomized controlled pilot trial with a 1:1 allocation ratio. Participants are stratified by sex (male/female) and randomized to either (1) a control group receiving generic sleep hygiene education or (2) an experimental group receiving personalized ML-derived behavioral recommendations.

The observation period is 60 days, which aligns with PSQI requirements for 30-day data collection periods. PSQI assessments are performed at (1) baseline (prior to device use), (2) day 30 (first monitoring period), and (3) day 60 (postintervention period). This enables subjective-objective correlation while establishing a baseline (days 1‐30) to be used as a covariate for intervention analysis (days 31‐60). While 30 days may be minimal for entrenching behavior change, this pilot will establish feasibility and preliminary effect sizes for future extended trials.

### Patient and Public Involvement

No patient or public involvement has been planned in the design, conduct, or reporting of this pilot trial owing to resource constraints for this project. Future definitive trials will incorporate patient and public involvement in intervention design, outcome selection, and dissemination strategies.

### Study Setting

This pilot study is conducted in Mexico City, a metropolitan area with over 21 million residents. Participants sleep in their habitual home setting while wearing a Samsung Galaxy Watch 4 continuously. All study interactions (consent, PSQI administration, device distribution, intervention delivery, and equipment return) occur in Mexico City through in-person meetings with a researcher.

### Eligibility Criteria

#### Eligibility Criteria Operationalization

The operationalization of the eligibility criteria is as follows:

- Sleep disorder screening: structured interview regarding diagnosed disorders, medications, and medical consultations; self-declaration (no PSG screening)
- Medical/psychiatric assessment: standardized questionnaires (no chart review)
- Nationality and age: birth certificate upon request
- Active workforce: full-time employment (≥40 hours/week) verified through documentation
- Limitations: self-report with documentation appropriate for a community-based pilot

#### Inclusion Criteria

The inclusion criteria are as follows: (1) Mexican nationality, (2) age 30‐60 years (minimizes age-related sleep physiology variability), (3) male or female gender, (4) active workforce status during the study, (5) residence in Mexico City during the study, (6) willingness and ability to use the wearable device for 60 days (at least during sleep), and (7) consent for providing sensitive personal data for the experiment.

#### Exclusion Criteria

The exclusion criteria are as follows: (1) diagnosis of chronic sleep disorders (eg, insomnia) requiring specific treatment, (2) major psychiatric or neurological conditions affecting sleep, (3) substance abuse or significant medical conditions impacting sleep quality, and (4) inability to comply with study requirements (eg, wearing the smartwatch).

### Recruitment: Participant Recruitment and Screening

The recruitment methods are as follows: (1) physical (study invitations posted on university and public hospital bulletin boards), (2) digital (study invitations on professional networking platforms like LinkedIn), and (3) referral (snowball sampling through professional networks).

### Screening Process

The screening process begins with initial contact from interested participants. Structured remote interviews are then conducted to screen participants based on the inclusion and exclusion criteria. Only participants meeting all criteria proceed to an in-person meeting for informed consent and verification of eligibility documentation. Retention leverages the intrinsic motivation of participants for sleep improvement, along with structured touchpoints at the 3 assessments.

### Interventions

#### Control Intervention: Generic Sleep Hygiene Education

Control group participants receive standardized sleep hygiene education on day 30, covering general recommendations applicable to all adults. Examples include maintaining a consistent sleep-wake schedule and sleeping between 7 and 9 hours per night. These recommendations are not personalized to individual sleep patterns.

#### Experimental Intervention: Personalized ML-Derived Recommendations

Experimental group participants receive personalized behavioral recommendations from a predefined library (Multimedia Appendix 1) on day 30 based on individual ML model analysis of their baseline sleep data (days 1‐30).

### ML Framework for Personalized Interventions

Regarding data structure and variables, Samsung Galaxy Watch 4 generates 1 target variable (sleep_score) and 10 explanatory variables (sleep_duration, movement_awakening, sleep_cycle, mental_recovery, physical_recovery, efficiency, Transition_N1, Light_N2, Deep_N3, and REM) per sleep session. Through preprocessing, 5 additional features are engineered (real_total_sleep, Transition_pct, Light_pct, Deep_pct, and REM_pct), and temporal (Night_Number) and identifier (Participant_ID) variables are added, creating an 18-column dataset. For ML analysis, 15 variables are used as ML input variables (excluding sleep_score as the target, and Participant_ID and Night_Number as nonpredictive identifiers).

The ML framework serves as the mechanism for generating personalized interventions, not as the study outcome. We use established methods (eg, random forest and SHAP [Shapley Additive Explanations]) documented in ML literature to identify each participant’s top predictive sleep parameters. Our innovation is applying these tools to generate behavioral recommendations from consumer device data, not developing new algorithms.

### Model Architecture and Development

The 15 Samsung Galaxy Watch 4 explanatory variables and additional engineered features serve as the base dataset. The training-validation framework involves a temporal split using baseline period data (days 1‐30) with a 70/30 split for training/validation, cross-validation through 5-fold time-series cross-validation to prevent data leakage, and individual modeling for separate models trained for each participant. Models include random forest regression (primary model for interpretability), gradient boosting (extreme gradient boosting [XGBoost]) for comparison, and multiple linear regression as baseline, with selection based on cross-validation root mean square error and R². Feature importance and selection involve SHAP values for feature importance ranking, recursive feature elimination to identify the top 5 predictors per participant, and correlation analysis to avoid multicollinearity (threshold: *r*>0.8). Personalized recommendation generation is based on individual feature importance ranking, and participants receive targeted recommendations from a predefined library (Multimedia Appendix 1), with a focus on their top 2‐4 modifiable sleep parameters.

### Intervention Delivery and Verification

Experimental group participants receive personalized recommendations explained by the researcher in person on day 30. Participants can clarify and provide feedback. A closure interview at day 60 verifies engagement and implementation attempts, and includes a subjective effectiveness assessment.

### Personalized Sleep Intervention Framework

The intervention content is based on individual ML feature importance ranking, and participants receive nonpharmacological recommendations from a predefined library (Multimedia Appendix 1) targeting their top 2‐4 predictive sleep parameters [108-116]. Example recommendations for TST optimization (if sleep_duration is a key predictor) are as follows: ensure wake-up time commitment for too much sleep and bedtime commitment for too little sleep. Example recommendations for sleep efficiency enhancement (if movement_awakening is a predictor) are as follows: use white noise, ensure a quiet environment, and restrict caffeine intake (avoid intake ≤6 hours before bedtime). Example recommendations for sleep timing regulation (if sleep_cycle is a predictor) are as follows: ensure a consistent sleep-wake schedule and keep the bedroom cool (around 18 °C‐20 °C).

Experimental group participants receive personalized recommendations, and the top 2‐4 recommendations are explained by the researcher during the in-person meeting. Adherence tracking involves self-reported compliance assessed at study closure (0%‐100% scale). Control group participants receive generic sleep hygiene education without personalization by the researcher during the in-person meeting.

### ML-Derived Heterogeneity by Design

Personalized interventions intentionally create between-participant variability within the experimental group, and this is the intervention’s defining feature, not a statistical flaw. Participant A might receive recommendations targeting REM sleep and sleep timing, while participant B might receive advice on deep sleep and wake episodes, based on individual ML model findings. This heterogeneity is the core innovation: targeting individual-specific parameters rather than universal protocols.

With regard to statistical implications, within-group variance in the experimental arm may exceed variance in the control arm. This reduces statistical power but reflects real-world personalization implementation. If personalization is effective, the increased precision of targeting individual needs should overcome variance increases. If personalization shows no benefit despite this heterogeneity, the approach is not superior to simpler generic advice. We do not attempt to statistically “control for” intervention heterogeneity, as it represents the intervention. Exploratory analyses will descriptively examine whether certain recommendation types (eg, duration-focused vs architecture-focused recommendations) show differential effectiveness, informing future research on personalization boundaries.

### Intervention Modifications

No modifications to assigned interventions are permitted during the 60-day study period. If participants cannot implement specific recommendations due to unforeseen circumstances, this is documented as nonadherence rather than modification. Adherence tracking captures whether participants attempted to follow recommendations, not whether recommendations were modified.

### Concomitant Care Protocol

The aspects related to the concomitant care protocol are as follows:

- Permitted: regular medical care, stable chronic disease management, and emergency care
- Prohibited: new sleep medications, sleep clinic consultations, or other sleep apps or programs
- Documentation: weekly medication diary and immediate reporting of treatment changes
- Safety: participants with sleep deterioration (<30 score for >1 week) withdrawn and referred
- Baseline: usual care patterns documented at enrollment

### Adherence Assessment

In the experimental group, adherence to personalized recommendations is assessed through self-report at the day 60 closure interview. Participants rate adherence on a scale from 0% to 100% for each recommendation provided. In the control group, adherence to generic sleep hygiene is assessed in a similar manner. Device wear adherence is monitored through objective data. Participants with missing data for >20% of nights may be excluded and replaced.

### Outcomes

#### Primary Outcome: Mean Samsung Galaxy Watch 4 Sleep Score During the Postintervention Period

We are working within proprietary constraints. We use the Samsung sleep score (1-100 scale) as the primary outcome for the following reasons: (1) real-world relevance: the score is the metric users see and seek to optimize in consumer wearable ecosystems; (2) continuous sensitivity: the scoring scale (1‐100) detects individual-level changes with precision, which categorical systems cannot achieve; (3) composite integration: the score algorithmically combines multiple sleep dimensions (duration, architecture, efficiency, and fragmentation), providing a holistic optimization target; (4) feasibility for personalization: continuous data for 60 nights enable individual ML models to identify predictive patterns; and (5) validation through correlation: secondary PSQI outcomes confirm that objective score improvements correspond to subjective sleep quality perception.

There are some limitations. The use of a proprietary algorithm limits the reproducibility of exact score computation. Moreover, there is no PSG validation of the composite score specifically (though component parameters are validated [36-43]). Furthermore, the manufacturer-specific metric precludes direct cross-device comparisons. These limitations are inherent to consumer wearable research and do not invalidate the personalization methodology (they contextualize the interpretation). Our framework is designed to work *within* these pragmatic constraints while remaining transferable to other manufacturers’ proprietary systems.

#### Secondary Outcome: PSQI Global Score Measured at Three Time Points

The PSQI (0-21 scale) validates that objective sleep score improvements correspond to the subjective perception of sleep quality and functional impact, ensuring clinical relevance from the patient perspective. The PSQI is not used as a competing primary measure but rather as a tool to provide complementary validation that device-measured changes reflect meaningful improvements in lived experience.

### Measurement Time Points

The measurement time points are as follows:

- Baseline (day 0): PSQI
- Days 1-30 (baseline period): continuous Samsung score collection for ML model training and baseline covariate establishment
- Day 30: PSQI; intervention delivery of personalized behavioral recommendations to the experimental group or intervention delivery of standardized sleep hygiene education covering general recommendations applicable to all adults
- Days 31‐60 (postintervention period): continuous Samsung score collection for the primary outcome
- Day 60: PSQI; closure interview

### Exploratory Outcomes

The exploratory outcomes include (1) individual sleep parameter changes (which specific variables improve), (2) adherence to personalized recommendations (self-reported 0%‐100% scale), and (3) behavioral confounders (alcohol consumption, exercise, and stress documented in the closure interview).

### Participant Timeline

**Figure 1.**
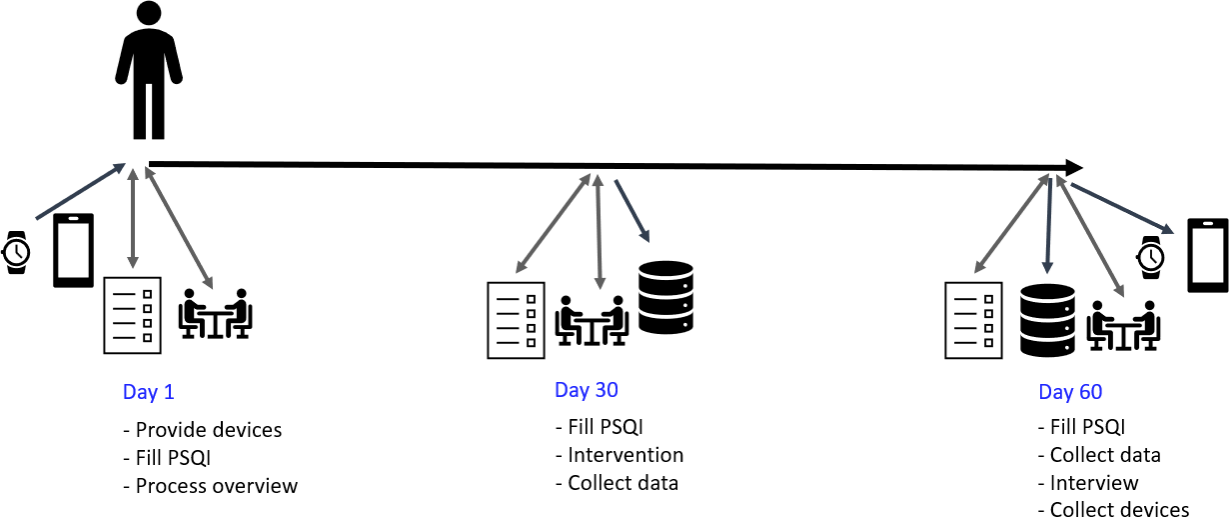
Illustration of the key actions during the 60-day study period in the control group. PSQI: Pittsburgh Sleep Quality Index.

The measurement time points after eligibility screening, informed consent approval, and randomization in the control and experimental groups are presented in Figures1  2, respectively.

**Figure 2.**
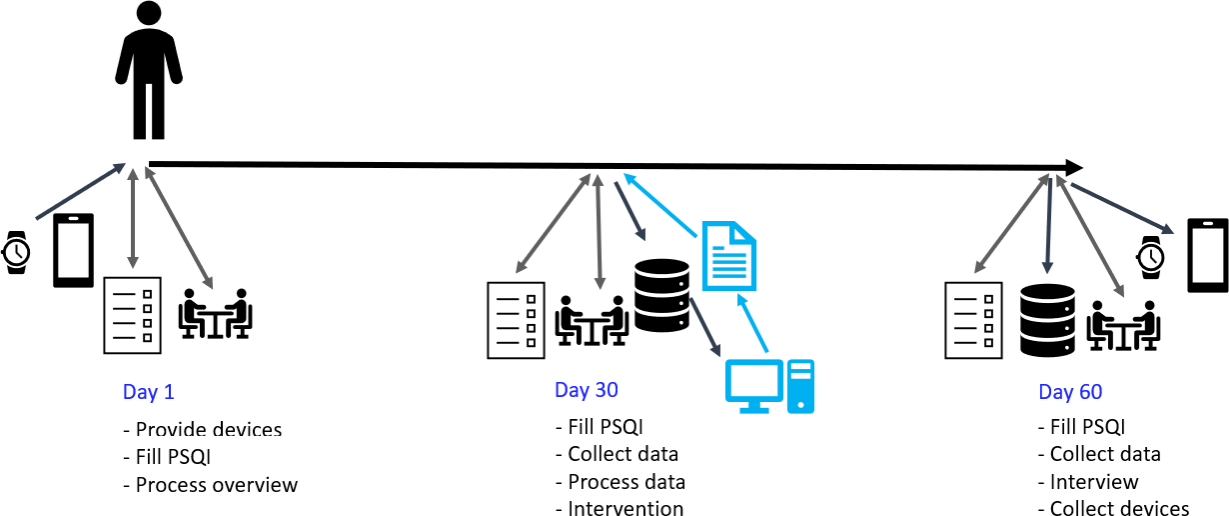
Illustration of key actions during the 60-day study period in the experimental group. PSQI: Pittsburgh Sleep Quality Index.

### Sample Size: Pilot Feasibility Justification

This pilot trial enrolls 32 participants (16 per arm; stratified by sex), and this is consistent with established pilot study guidelines recommending 12‐15 participants per arm for feasibility assessment [117-120]. The sample size is explicitly designed for pilot objectives, not definitive efficacy testing.

### Pilot Objectives

The pilot objectives are as follows:

1. Feasibility: Can we successfully recruit, enroll, randomize, and retain participants? Can personalized ML interventions be delivered? Do participants engage with recommendations?
2. Preliminary effect size estimation: What magnitude of difference between groups might we observe? What variability exists within groups? What baseline-to-follow-up correlation informs covariate adjustment? The estimates can inform future randomized controlled trial (RCT) sample size calculations.
3. Methodology refinement: What challenges arise in data collection, ML model training, and intervention delivery? What proportion of nights yield usable data? Which sleep parameters show the most predictive value?
4. Hypothesis generation: Does personalization show a promising signal warranting a larger trial? Do certain participant subgroups respond better?

### Power Analysis Context: Not Designed for Definitive Testing

The sample size provides adequate power (>80%) only for very large effects (*d*≥1.0). For moderate effects typical in sleep research (*d*=0.3‐0.7), the power is insufficient, which is consistent with this study’s role as a feasibility-focused pilot trial. We explicitly acknowledge insufficient power for moderate effects. The power analysis table showing power for different effect sizes is presented in Multimedia Appendix 2. If moderate-to-large effects exist, we may detect a signal, and if effects are small, this pilot will yield null results, informing researchers that larger samples, longer interventions, or different approaches are needed. Both outcomes are valuable for the advancement of the field.

### Sample Size for a Future RCT

Based on pilot effect sizes and variability estimates, a future definitive RCT would require approximately 175 participants per arm (350 participants in total) to detect moderate effects (*d*=0.5), considering 80% power, *α* of .05, and 15% attrition. Pilot findings will refine these projections.

### Assignment of Interventions (Allocation)

Sequence generation is performed through computer-generated randomization using Python (numpy.random.RandomState with seed=42 for reproducibility). Participants are stratified by sex (male/female) to ensure balance. Block randomization with randomly permuted block sizes (2, 4, 6) is used to maintain unpredictability while ensuring approximately equal group sizes throughout recruitment. The approach is implemented using numpy, hashlib, and pandas libraries.

### Allocation Concealment Mechanism

The randomization sequence is stored in a password-protected file accessible only to the researcher. Allocation is revealed only after the participant completes the baseline PSQI and receives the device (day 0), preventing selection bias. Participants are enrolled and allocated in cohorts of 2‐8 to maintain seasonal balance across the 15-month recruitment window.

### Implementation

The researcher generates an allocation sequence and assigns interventions according to the sequence concealed in sealed, sequentially numbered envelopes, which are opened only at the allocation time point. Participants are informed of group assignment on day 30 (intervention delivery) for the experimental group or at close-out for the control group.

### Seasonal Control

To account for seasonal or environmental factors over the 15-month recruitment window, device assignment ensures equal representation from both groups at each time point. An even number of participants receive devices simultaneously, preventing differential seasonal effects between groups.

### Blinding (Masking) Level

This is an open-label pilot trial with partial blinding where feasible. Participants become aware of group assignment at different time points (experimental group participants are informed on day 30 at intervention delivery; control group participants are informed on day 60 at close-out). Samsung Galaxy Watch 4 automatically records objective sleep data independent of participant or researcher knowledge, eliminating measurement bias for the primary outcome. The researcher analyzing primary outcome data (Samsung scores) remains blinded to group allocation, and unblinding occurs on day 30 to identify participants for analysis in the experimental group in order to provide personalized recommendations.

### Justification for Partial Blinding

Complete participant blinding is impossible for providing personalized and generic advice. The primary outcome (device-recorded sleep score) has objective measurement protection. For the secondary outcome (PSQI), assessor blinding is attempted where feasible. This pragmatic approach balances methodological rigor with pilot study feasibility.

### Data Collection Quality Control

The following measures are implemented for user-related issues: (1) noncompliance: participants with missing data for >20% of nights are excluded and replaced; (2) charging issues: backup chargers are provided, and daily battery monitoring is required; (3) unauthorized modifications: settings are locked, and violations lead to replacement; and (4) sync delays: daily commitment for cloud backup within 24 hours. The following measures are implemented for device-related issues: (1) fragmented recording: nights without combined scores are excluded; (2) data mismatch: nights with artifact conflicts are excluded; and (3) sync errors: multiple attempts are made with escalating intervals. For sensor inaccuracies, quality control is performed through sleep diaries, and outliers trigger replacement. Excluded participants are replaced from the recruitment pool to maintain the sample size. The expected night exclusion impact is around 18%, with anticipated replacement ensuring the target sample.

Subjective data collection is performed through PSQI forms completed on paper. Information is manually entered by the researcher into a password-protected spreadsheet with self-verification (data are entered twice by the researcher to ensure accuracy). Data are processed through the manufacturer’s algorithms to interpret the findings of the 10 explanatory variables and the target variable (Table 1).

**Table 1.** Target and explanatory variables collected by the wearable (indispensable for this study).

Variable		Description	Unit	Data type
Explanatory variable				
sleep_duration		Sum of N1, N2, N3, and REM^a^	Minutes	Integer
movement_awakening		Count of movement during sleep	Number	Integer
sleep_cycle		Count of sleep cycles	Number	Integer
mental_recovery		Score assigned according to the REM phase	Score (0‐100 scale)	Integer
physical_recovery		Score assigned according to the N3 phase	Score (0‐100 scale)	Integer
efficiency		real_total_sleep by sleep_duration	Percentage	Integer
Transition_N1		Sum of minutes of sleep in the N1 phase	Minutes	Integer
Light_N2		Sum of minutes of sleep in the N2 phase	Minutes	Integer
Deep_N3		Sum of minutes of sleep in the N3 phase	Minutes	Integer
REM		Sum of minutes of sleep in the REM phase	Minutes	Integer
Target variable				
sleep_score		Score assigned for the night	Score (1‐100 scale)	Integer

a REM: rapid eye movement.

### Data Preprocessing Pipeline

Data are downloaded from Samsung Cloud onto the participant’s mobile device for transfer to the researcher’s computer for analysis. Moreover, individual nights are processed, sleep stages are integrated, and participant IDs are assigned. For quality control, nights with missing or corrupted segments are excluded, fragmented sleep data without combined scores are removed, daytime sleep (outside 7 PM to 11 AM) is excluded, nights with ≥30 minutes of stage/total discrepancies are excluded, and outliers >3 SD from baseline are flagged. For feature engineering, additional features (sleep stage percentage and real TST) are derived from the 10 device variables in ML analysis. Completeness of reporting is ensured by documenting the percentage of excluded nights and the reasons for exclusion.

### Data Management

Data entered and collected by Samsung Galaxy Watch 4 are automatically synced to Samsung Cloud (encrypted transmission) upon pairing. The PSQI forms are completed on paper, and the information is manually entered by the researcher into a password-protected spreadsheet with self-verification (data entered twice by the researcher to ensure accuracy). All data stored on the researcher’s local computer are encrypted, providing additional privacy protection through local-only storage. Moreover, data are stored in 2 separate local files as a backup measure for protection against file corruption. Access control is set, with only the researcher having access to all study data, including identifiable information linking Participant_ID to personal information. No other personnel have access to the data.

Data security involves (1) physical security (paper PSQI forms are stored in a locked filing cabinet at a secure location), (2) digital security (electronic files are password-protected and encrypted, and are stored on the researcher’s local computer only [no remote or cloud storage of identifiable data]), and (3) transmission security (Samsung device data download uses encrypted protocols [HTTPS]).

### ML-Specific Data Security Measures

ML model training is conducted on the researcher’s local computer with no internet connectivity during sensitive data processing to prevent data exfiltration. All access to ML model files and training data is logged within the researcher’s system for accountability. Cross-validation and model testing use separate data partitions, and there is no test data contamination of training sets. Validation results are stored separately from production models on the researcher’s local system. No commercial ML platforms (eg, AWS SageMaker and Google AutoML) are used to prevent corporate access to participant data. All processing uses open-source libraries (scikit-learn, XGBoost, and SHAP) on local infrastructure.

Data quality assurance includes data completeness checks (missing nights and sync failures), value range checks for physiologically implausible values, and analysis data validation using scripts for checking distributions, outliers, and duplicates.

A retention and destruction policy is implemented. Identifiable data will be retained for 5 years after publication, and then, the data will be securely destroyed (paper shredding and secure overwriting of electronic files). Deidentified data will be retained indefinitely.

### Statistical Methods

#### Primary Analysis: Single-Covariate Analysis of Covariance

The mean sleep score in the postintervention period (days 31‐60) is compared between groups using analysis of covariance with a single covariate: mean sleep score in the baseline period (days 1‐30).


$$Model:Postintervention\;score=\beta_{0}+\beta_{1}\;(group)+\beta_{2}\;(baseline\;score)+\epsilon$$

The control group is coded as “0,” and the experimental group is coded as “1.” The primary inference is based on the β₁ coefficient (group difference adjusted for baseline). The effect size is calculated as Cohen *d* with 95% CIs. Statistical significance is assessed at *α*=.05 (1-tailed per directional hypothesis), but the interpretation emphasizes the effect size magnitude and CI width, given the pilot nature of the study.

We do not use multiple covariates (age, sex beyond stratification, and behavioral factors) to avoid overfitting with the small sample size. Sex stratification in randomization addresses balance concerns without requiring covariate adjustment.

#### Secondary Analysis: PSQI Changes

The PSQI global score change from baseline (day 0) to follow-up (day 60) is compared between groups using the independent samples *t* test. The correlation between the Samsung score change and PSQI change is calculated using Pearson *r*.

#### Exploratory Analyses: Hypothesis Generation Only

The exploratory analyses involve the following three aspects:

1. Individual parameter changes: Compare the change in specific sleep variables (duration, deep sleep percentage, and REM percentage) between groups using *t* tests (unadjusted for multiple comparisons, given the exploratory nature).
2. Adherence subgroups: In the experimental group, compare outcomes between high (≥80%) and low (<80%) self-reported adherence using the Mann-Whitney *U* test.
3. Predictive feature patterns: Perform a descriptive analysis of which sleep parameters are most frequently identified as the top predictors across participants.

All exploratory analyses are labeled hypothesis-generating, not confirmatory. No adjustments are made for multiple comparisons, given the pilot scope of the study. Regarding the handling of missing data for the primary outcome (sleep score), participants are excluded if sufficient night data are not available in the baseline and postintervention periods, and action on the secondary outcome (PSQI) is derived from the handling of the primary outcome. This protocol contains the complete statistical analysis plan, and no separate statistical analysis plan exists.

### Interpretation Guidance

All findings will be interpreted as preliminary, hypothesis-generating signals requiring confirmation in adequately powered trials. We will report effect sizes, CIs, and descriptive patterns rather than definitive claims of efficacy or lack thereof. The pilot’s value lies in feasibility demonstration and effect size estimation for future sample size calculations, not in definitive answers about intervention effectiveness.

### Data Monitoring Oversight

The researcher monitors study progress, enrollment rates, data quality, and adverse events throughout the study period. No formal data monitoring committee is established, given the pilot scale of the study and the use of a low-risk intervention (behavioral recommendations only).

### Interim Analysis

No formal interim analysis is planned. The researcher conducts data quality monitoring but does not analyze outcomes until study completion to prevent bias.

### Stopping Guidelines

The study may be stopped early if (1) enrollment fails to reach the target within 18 months, (2) 50% of enrolled participants withdraw, (3) serious adverse events attributable to study procedures occur, or (4) unexpected device failures affect >30% of participants.

### Harms: Adverse Event Tracking

The following events are tracked:

- Study-related events: documented at 3 interaction points (baseline, day 30, and day 60)
- Behavioral or environmental factors: captured in the closure interview
- Withdrawal events: managed according to informed consent procedures
- All adverse events: recorded and reported in the final analysis

### Behavioral Factor Assessment

The study closure interview captures key behavioral factors affecting sleep (alcohol consumption, exercise, and stress) that may confound the results. Rather than excluding participants with these factors, we document them to understand their impacts on intervention effectiveness. This approach maintains ecological validity while acknowledging potential confounders.

### Instruments

#### Commercial-Grade Wearables

The study uses Samsung Galaxy Watch 4 [27] for sleep tracking. Samsung Galaxy Watch 4 has been selected following a systematic evaluation of available consumer sleep tracking devices. The selection criteria are as follows: validation evidence (published PSG comparison studies), parameter comprehensiveness (10 sleep variables vs 3‐5 in most competitors), data accessibility for research use, cost feasibility (US $200‐250 per unit [within budget]), operationalization of sleep quality through a composite sleep score (scale 1-100) that algorithmically integrates multiple dimensions, and user compliance factors (battery life >24 hours). While alternatives exist (Fitbit: limited architecture data; Apple Watch: iOS requirement; Garmin: higher cost; WHOOP: subscription model), Samsung Galaxy Watch 4 provides an optimal balance of scientific validity, data richness, and practical feasibility for this pilot study [29,30,36-43,71,105-107].

This sleep monitoring system is noninvasive, involves ambulatory or home-based deployment, is contact-based (wrist-worn), has comprehensive architecture analysis parameters, involves real-time analysis, and is a consumer-grade device with clinical validation studies. The sensors include an accelerometer, a gyroscope, a photoplethysmography sensor, a bioelectrical impedance sensor, and an ambient light sensor [27,121]. This positioning offers ecological validity while acknowledging trade-offs in precision compared with laboratory approaches.

#### Pairing Devices

Mobile phones (1 per wearable) are used for data synchronization.

#### PSQI Questionnaire

The PSQI is used for evaluation. It is a validated subjective sleep quality assessment [28].

#### Computer

The researcher’s computer is used for data storage, processing, and ML analysis.

### Study Timeline

The overall experimentation period is 15 months (all volunteers). The start date is October 4, 2024, and the maximum end date is December 31, 2025. The experiment duration for each volunteer is 60 days.

### Artificial Intelligence Accountability and Algorithmic Governance

This study uses ML algorithms to analyze participant sleep data and generate personalized recommendations. The governance framework is presented below.

#### Algorithmic Transparency Obligations

##### Participant Information

The participant information aspects are as follows:

- Informed consent explicitly states the use of ML for sleep data.
- Participants are informed that recommendations represent statistical pattern recognition, not clinical judgment.
- Models identify correlations, not causation.
- Recommendations are behavioral suggestions, not medical advice.
- Participants maintain full autonomy to accept, modify, or ignore recommendations.

##### Explainability

The explainability aspects are as follows:

- ML recommendations are accompanied by plain-language explanations.
- SHAP visualizations show which sleep parameters influence the individual model.
- Example: “Your model identified REM sleep and sleep timing consistency as your two strongest predictors. When you had ≥90 minutes REM and went to bed within 30 minutes of your average time, your scores were typically 8‐12 points higher.”

##### Human Oversight

The human oversight aspects are as follows:

- ML models suggest recommendations, but the principal researcher reviews all outputs before delivery.
- The human-in-the-loop approach ensures recommendations are safe, coherent, and appropriate.
- Models do not make automated health decisions.

### Participant Rights Concerning Algorithmic Outputs

Participant rights are as follows:

- Right to explanation: Participants may request a detailed explanation of how personalized recommendations are generated.
- Right to question: Participants may challenge recommendations they believe are inaccurate or inappropriate. The researcher is available for discussion and can modify recommendations based on participant feedback.
- Right to decline: Participants may decline to implement some or all ML-derived recommendations without penalty or withdrawal from the study. Nonadherence is tracked but does not affect participant standing.
- Right to human review: Participants may request that the principal researcher personally review their data and provide alternative recommendations if dissatisfied with ML outputs.
- Access to model information: Participants may request information about ML methodology (algorithms used, training process, and validation approach) in plain language.

### Data Retention and Security for ML Processes

The data retention and security aspects are as follows:

- Raw sleep data: Data from 1920 nights are stored encrypted on the researcher’s local computer with password protection and backup. The data are retained for 5 years after publication and are then securely destroyed.
- ML model files: Individual participant models (random forest and XGBoost) are stored on the researcher’s local computer separately from identifiable data. Models contain learned patterns but no raw data. The information is retained indefinitely for reproducibility verification. The information can be deleted upon participant request during the identifiable data retention period.
- SHAP values and feature importance rankings: The data are stored as CSV files on the researcher’s local computer, linking Participant_ID to rank predictive features. These data have the same retention schedule as the raw data (5 years after publication).
- Transmission security: No ML processing occurs on participant devices or cloud services. All analyses are conducted on the researcher’s local computer. No data are transmitted to third parties or commercial ML platforms.

### Accountability Framework

The accountability framework involves the following aspects:

- Adverse events from artificial intelligence (AI): If ML-derived recommendations cause participant harm (eg, a recommendation to reduce sleep duration leads to excessive daytime sleepiness affecting work safety), the event is documented as an adverse event. The researcher notifies the ethics committee, and an investigation is conducted to determine if a systematic model error exists.
- Replication and audit: The ML code, trained models, and analysis scripts are made available to the ethics committee and peer reviewers upon request.
- Model performance monitoring: During the study, the principal researcher monitors whether the experimental group shows unexpected negative outcomes (eg, worsening sleep scores), triggering a review of ML recommendation patterns for systematic errors.

### Compliance With AI Governance Frameworks

This study follows the principles from the World Health Organization (WHO) guidance on ethics and governance of AI for health (transparency, accountability, and human oversight) and the International Association of Scientific, Technical & Medical Publishers recommendations for AI use in academic manuscript preparation [122].

### Ethical Considerations

#### Research Ethics Approval

This research has been reviewed and approved by the Institutional Committee on Research Ethics of Tecnológico de Monterrey (ethics approval number: CA-EIC-2408-06; approval date: October 2024; review type: full committee review). The study complies with the principles of the Declaration of Helsinki for medical research involving human participants and the international ethical guidelines for health research. There is no compensation for participation in the study.

#### Protocol Amendments

Any modifications to the eligibility criteria, interventions, outcomes, or ethical procedures will be submitted to the ethics committee for review and approval prior to implementation. Amendments will be documented in protocol version history with the rationale for changes.

#### Informed Consent Collection

The principal researcher has completed ethical training to obtain informed consent from potential trial participants.

#### Additional Consent Provisions for the Collection and Use of Participant Data and Biological Specimens in Ancillary Studies

It is not applicable to consider additional consent provisions for the collection and use of participant data and biological specimens in ancillary studies since there are no additional studies.

#### Confidentiality: Data Protection

Personal data are not shared. The data are only used to allocate participants to the study groups and to ensure consistent assignment of the proper Participant_ID to keep the process accurate and deidentified. The participants can access their personal data or request data correction. Data are protected before, during, and after the trial.

#### Compensation for Research-Related Injury

In the unlikely event of research-related injury, immediate medical care coordination will be provided by the researcher, participants can use existing health insurance for treatment, no additional compensation will be provided, and the incident will be documented and reported to the ethics committee. The anticipated risk is minimal (behavioral intervention, noninvasive device use, skin irritation from the device, sleep tracking anxiety, and time commitment).

### Ancillary and Posttrial Care

#### Incidental Findings

Participants with incidental findings during the study may be withdrawn and referred for severe sleep deterioration (sleep score <30 for >1 consecutive week). Participants may be withdrawn from the study and referred to sleep medicine services at their medical institutions. Contact information may be provided for sleep specialists in Mexico City. There are no study-related costs for referral (participants use existing health insurance).

#### Posttrial Care

There is no posttrial provision of interventions. Personalized recommendations are study-specific research interventions, not ongoing clinical care interventions. After study completion, participants may continue using strategies that helped them. There is no ongoing monitoring or intervention delivery. Participants are encouraged to consult health care providers for persistent sleep concerns. No direct medical benefits are guaranteed.

### Access Control and Protection Layers for Potential and Excluded Participants

Data collected from potential participants who do not fill out the informed consent form (Multimedia Appendix 3) or the commitment letter (Multimedia Appendix 4) and those who voluntarily withdraw or need to be excluded from the study are immediately and securely deleted.

### Access Control and Protection Layers for Enrolled Participants

Only the principal researcher has access to all study data, including identifiable information linking Participant_ID to personal information (mapping). The only risk is the mapping between the participant and the alias, which is kept in physical form at a secure physical location. Accessing it through a digital hacking attack is not possible. Participant data are stored securely and can be accessed only by authorized research personnel. Data will be retained for 5 years after publication, while the deidentified dataset will be retained indefinitely.

Data security involves (1) physical security (paper PSQI forms are stored in a locked filing cabinet at a secure location), (2) digital security (electronic files are password-protected and encrypted, and are stored on the researcher’s local computer only [no remote or cloud storage of identifiable data]), and (3) transmission security (Samsung device data download uses encrypted protocols [HTTPS]).

### Anonymization Process (Deidentification)

Anonymization involves assigning an alias to the personal data collected from each participant at different process steps. Email or Samsung accounts do not use personal data. These accounts are only used for data collection, as the approach is already anonymous by design.

An alias is used at each phase of the process. The smartwatch user setup is generic, and the smartwatch collects objective data without the participant’s personally identifiable data. The same device and setup are used throughout the study. The pairing device setup, like the user setup, is generic, and thus, the mobile phone collects objective data without the participant’s personally identifiable data. PSQI questionnaire data collection uses the Participant_ID alias for subjective data collection without the participant’s personally identifiable data. In the last phase, the data are merged into a single dataset, which is one of the research outputs.

### ML and AI Elements

Participants receive an explanation that the algorithms will analyze individual sleep patterns; a clarification that recommendations are statistically derived suggestions, not medical diagnoses; a statement that they retain full autonomy to follow or ignore recommendations; a description of SHAP explainability (plain-language explanations of personalized insights); and an assurance of human oversight (the researcher reviews all ML outputs before delivery).

### Enhanced AI/ML-Specific Consent Elements

Given the ML component, participants are explicitly informed about how ML models work (pattern recognition in their individual data) and what SHAP analysis means (identifying which factors predict their sleep scores). They are also informed that recommendations are correlational, not causal (eg, “REM correlates with higher scores” not “REM causes better sleep”), and that they have a right to question or decline any recommendation. Furthermore, they are told that the researcher reviews the recommendations before delivery (human-in-the-loop approach) and that no automated health decisions are made.

## Results

This protocol describes the planned analytical approach. The actual results, including group comparisons, effect sizes, statistical significance levels, and interpretation of findings, will be reported in a subsequent manuscript following study completion. The study is funded by Instituto Tecnológico y de Estudios Superiores de Monterrey (publishing fee) and *Secretaría de Ciencia, Humanidades, Tecnología e Innovación*, which was formerly called *Consejo Nacional de Ciencia y Tecnología* (wearable and pairing device equipment funding). The funding reference number is REF4661568-251121 (November 24, 2025). The trial has not yet been registered, but we intend to register it on ClinicalTrials.gov.

The study is currently in progress. Recruitment started in August 2024 and ended in July 2025. Data collection is expected to be completed by December 2025. The study will (1) compare 960 nights from the control group with 960 nights from the experimental group to explore whether ML interventions can improve sleep scores using wearable technology, (2) generate a dataset from objective data for iterative model training and analysis, (3) correlate objective and subjective sleep quality metrics, and (4) establish whether a feasible framework for proactive sleep quality approaches can be developed. The final analyzed results incorporating all quality control procedures, statistical analyses, and exploratory investigations will be available by March 2026, and the findings will be submitted for peer-reviewed publication within 6 months of study completion.

Upon completion of data collection by December 2025, the study will generate a comprehensive dataset from 32 participants (16 per arm) wearing Samsung Galaxy Watch 4 devices for 60 consecutive nights each, yielding approximately 1920 nights of sleep data. The final dataset will include 18 variables (Table 2).

**Table 2.** Final variables included in this study.

Variable		Description	Unit	Data type
Explanatory variable				
Participant_ID		Unique number assigned to each participant	Number	Integer
Night_Number		Night count	Number	Integer
sleep_duration		Sum of N1, N2, N3, and REM^a^	Minutes	Integer
movement_awakening		Count of movement during sleep	Number	Integer
sleep_cycle		Count of sleep cycles	Number	Integer
mental_recovery		Score assigned according to the REM phase	Score (0‐100 scale)	Integer
physical_recovery		Score assigned according to the N3 phase	Score (0‐100 scale)	Integer
efficiency		real_total_sleep by sleep_duration	Percentage	Integer
Transition_N1		Sum of minutes of sleep in the N1 phase	Minutes	Integer
Light_N2		Sum of minutes of sleep in the N2 phase	Minutes	Integer
Deep_N3		Sum of minutes of sleep in the N3 phase	Minutes	Integer
REM		Sum of minutes of sleep in the REM phase	Minutes	Integer
real_total_sleep^b^		Sum of N2, N3, and REM	Minutes	Integer
Transition_pct		Percentage of sleep in the N1 phase	Percentage	Float
Light_pct		Percentage of sleep in the N2 phase	Percentage	Float
Deep_pct		Percentage of sleep in the N3 phase	Percentage	Float
REM_pct		Percentage of sleep in the REM phase	Percentage	Float
Target variable				
sleep_score		Score assigned for the night	Score (1‐100 scale)	Integer

a REM: rapid eye movement.

b The “real_total_sleep” variable excludes the N1 transition stage from total sleep duration, as this brief transitional period between wakefulness and consolidated sleep is typically excluded from restorative sleep calculations in sleep research.

The dataset will enable comparisons between the control group (960 nights; participants receiving generic sleep hygiene education on day 30) and the experimental group (960 nights; participants receiving personalized ML-derived recommendations on day 30).

Each night of sleep data will be automatically collected through sensors by Samsung Galaxy Watch 4 and processed through the manufacturer’s proprietary algorithms to generate objective sleep metrics. Data will be organized by participant identifier and temporal sequence to enable both group-level comparisons and individual-level ML model development.

Data completeness evaluation based on pilot testing and literature review suggests that approximately 18% of individual nights may be excluded due to the quality control criteria, with participant replacement ensuring that the final dataset achieves the target 1920 nights across all study participants.

## Discussion

### Proprietary Algorithm Constraints

The Samsung Galaxy Watch 4 sleep score is a proprietary composite metric with undisclosed algorithmic weightings. This limits reproducibility of the exact score calculation and precludes independent validation against PSG for the composite metric specifically (though component parameters have validation evidence [36-43]). We acknowledge these constraints openly and position our methodology as working within the pragmatic realities of consumer wearable research.

Our framework demonstrates that effective personalization can be generated despite algorithmic opacity through pattern recognition that transcends the need to understand underlying computations. If reducing prebedtime screen time correlates with a participant’s score improvements across 30 nights, that pattern remains actionable regardless of Samsung’s computational details. The methodology’s value lies in its transferability. Future studies can apply identical approaches to Fitbit, Apple Watch, Garmin, or other platforms, testing whether personalization generalizes across manufacturers.

### Potential Outcomes and Interpretation Framework

This pilot study is explicitly designed for feasibility assessment and preliminary effect size estimation, not definitive efficacy testing. As noted in the Methods section, this pilot has limited power for moderate effects. Pilot studies generate hypotheses and refine the methodology, and definitive answers require subsequent adequately powered trials.

### Study Limitations

This pilot study has several limitations that contextualize interpretation. The small sample (n=32) provides insufficient power for moderate effects and limits subgroup analyses. The 30-day intervention period may be inadequate for sustained behavior change. The single-device focus prevents cross-platform validation. Geographic and cultural limitations (Mexico City context with unique altitude, pollution, and lifestyle factors) constrain generalizability. The exclusion of sleep disorder populations limits applicability to healthy adults seeking optimization rather than clinical treatment.

The proprietary algorithm dependency limits reproducibility, though we position this as a pragmatic constraint our methodology addresses rather than an insurmountable barrier. Consumer-grade sensor accuracy has inherent limitations compared to PSG, potentially obscuring true intervention effects with small samples.

We do not collect comprehensive outcome data assessing daytime functioning, cognitive performance, mood, physical health markers, or quality of life. The focus remains on sleep score optimization without demonstrating whether improvements translate to meaningful real-world benefits. Future studies should incorporate broader outcomes to establish clinical relevance beyond device metrics.

### Study Strengths

Despite these limitations, this pilot study has several strengths. The manufacturer-independent framework provides a transferable methodology applicable across platforms, potentially driving the field toward less proprietary and more user-centered approaches. The dual validation combining objective and subjective measures addresses both physiological and patient-reported domains. The explicit pilot design appropriately positions findings as preliminary and hypothesis-generating rather than making premature efficacy claims. The comprehensive data governance framework (local-only storage, multilayer anonymization, and explicit AI accountability measures) provides a model for ethical wearable research. The transparent reporting approach with public data sharing enables replication and meta-analytic integration regardless of the findings.

### Implications for Future Research

If this pilot study demonstrates feasibility and promising effect sizes, subsequent research should prioritize the following: (1) an adequately powered definitive RCT (n=350 total) with extended intervention periods (3‐6 months); (2) concurrent PSG validation in subsamples to establish measurement precision; (3) multidevice validation testing framework generalizability across manufacturers; (4) comprehensive outcome batteries, including daytime functioning, cognitive performance, and quality of life; (5) cost-effectiveness analyses comparing personalized and generic approaches; and (6) implementation science examining real-world deployment barriers and facilitators.

The pilot dataset will enable iterative ML model refinement, identification of optimal baseline data collection periods (Is 30 days sufficient or should future studies collect data for 60‐90 days?), and exploration of which sleep parameters show the most reliable personalization effects. These methodological refinements will strengthen subsequent trials.

### Conclusions

This pilot study establishes the feasibility and preliminary evidence base necessary for future definitive trials testing whether ML personalization involving consumer wearable data can improve sleep outcomes. The manufacturer-independent framework, dual validation approach (objective device metrics plus subjective PSQI), and explicit pilot design appropriately position this study as hypothesis-generating research that could inform the next generation of personalized digital health interventions for sleep quality optimization in healthy populations.

The proposed approach tests a specific mechanistic hypothesis: precision targeting of high-impact parameters identified through individual ML models may enhance motivation through personalized predictions while reducing cognitive burden compared with generic recommendation lists. Rather than providing universal sleep hygiene advice, this methodology will generate interventions tailored to each participant’s unique sleep architecture patterns and predictive factors.

Building upon foundational work establishing wearable feasibility for sleep monitoring [36,71,105-107], this study’s planned contribution to the field involves testing whether ML-derived personalization improves outcomes compared with generic sleep hygiene. If the methodology proves feasible and preliminary effect sizes suggest a benefit, the resulting dataset of 1920 nights will enable iterative model refinement and provide evidence for scaling personalized sleep interventions to larger populations.

Several important considerations frame the interpretation of the anticipated findings. The study acknowledges the inherent limitations of consumer wearable research, particularly reliance on Samsung’s proprietary sleep score algorithm. While this limits reproducibility of exact score computation and precludes independent PSG validation of the composite metric, the personalization framework itself is deliberately manufacturer-agnostic. Additional methodological constraints include the single-device focus, the limited geographic and cultural generalizability (Mexico City context with unique altitude, pollution, and lifestyle factors), potential measurement errors inherent to consumer-grade sensors, and the 30-day intervention period, which may be insufficient for sustained behavior change in entrenched sleep habits. The study excludes individuals with diagnosed sleep disorders, limiting the findings to healthy populations seeking sleep quality optimization rather than clinical treatment.

If validated through this pilot study, the methodology could direct sleep optimization from universal protocols toward individualized behavioral targeting, offering a scalable approach to sleep health improvement.

However, if the findings reveal no difference between personalized and generic interventions, there would be valuable evidence that (1) generic interventions may be equally effective and more cost-efficient for healthy populations, (2) the ML personalization approach requires refinement before clinical utility can be established, or (3) consumer wearables may not yet provide sufficient data quality for meaningful pattern detection at the individual level. Both positive and null findings will advance scientific understanding and inform future research directions.

The results will be available by March 2026 and will be disseminated through a peer-reviewed publication regardless of the findings (positive, null, or negative). A deidentified dataset will be part of the study output. This transparency supports scientific rigor and allows the broader research community to build upon the methodological framework regardless of this pilot’s specific outcomes.

## Supplementary material

10.2196/76415 Multimedia Appendix 1 Predefined library.

10.2196/76415 Multimedia Appendix 2 Power analysis table showing power for different effect sizes.

10.2196/76415 Multimedia Appendix 3 Informed consent form.

10.2196/76415 Multimedia Appendix 4 Commitment letter.

10.2196/76415 Checklist 1 SPIRIT 2025 checklist.

## Abbreviations

AI: artificial intelligence
ML: machine learning
PSG: polysomnography
PSQI: Pittsburgh Sleep Quality Index
RCT: randomized controlled trial
REM: rapid eye movement
SHAP: Shapley Additive Explanations
SPIRIT: Standard Protocol Items: Recommendations for Interventional Trials
TIB: time in bed
TST: total sleep time
XGBoost: extreme gradient boosting
