# Supplementary material for: Personalized Machine Learning Intervention to Improve Sleep Quality Using Wearable Technology in Healthy Middle-Aged Adults From Mexico City: Protocol for a Pilot Randomized Controlled Trial
Source: JMIR Res Protoc. 2026 Jan 6;15:e76415. doi: 10.2196/76415 (PMC12773695; doi:10.2196/76415)
Supplement: Multimedia Appendix 1 [file resprot-v15-e76415-s001.pdf]

Prioritized Evidence-Based Sleep Hygiene Checklist by Category

TOTAL SLEEP TIME

Target: Achieve 7-9 hours of sleep

High Impact

1. **Consistency**: Same sleep/wake times daily (including weekends)
2. **Pre-sleep routine**: 30-60 minute wind-down period
3. **Screen time**: Avoid 1-2 hours before bed
4. **Evening light**: Dim lights 2 hours before bed

Moderate Impact

5. **Caffeine**: Avoid 6+ hours before bedtime
6. **Temperature**: Keep bedroom cool (around 65-68°F)
7. **Bedroom use**: Reserve for sleep and intimacy only
8. **Alcohol**: Limit evening consumption

Lower Impact

9. **Morning light**: Get bright light exposure upon waking
10. **Heavy meals**: Avoid 3+ hours before bed
11. **Nicotine**: Avoid, especially near bedtime
12. **Noise**: Use white noise or ensure quiet environment

SLEEP CYCLES

Target: Complete 4-5 full 90-120 minute cycles

High Impact

1. **Consistency**: Same sleep/wake times daily
2. **Temperature**: Keep bedroom cool (around 65-68°F)
3. **Alcohol**: Limit evening consumption (disrupts REM cycles)
4. **Light**: Minimize light exposure during sleep

Moderate Impact

5. **Screen time**: Avoid 1-2 hours before bed
6. **Evening light**: Dim lights 2 hours before bed
7. **Caffeine**: Avoid 6+ hours before bedtime
8. **Noise**: Use white noise or ensure quiet environment

Lower Impact

9. **Pre-sleep routine**: 30-60 minute wind-down period
10. **Heavy meals**: Avoid 3+ hours before bed
11. **Nicotine**: Avoid, especially near bedtime
12. **Bedding**: Comfortable mattress and pillows

MOVEMENTS AND AWAKENINGS

Target: Minimize sleep disruptions

High Impact

1. **Noise**: Use white noise or ensure quiet environment
2. **Light**: Minimize light exposure, use blackout curtains
3. **Temperature**: Keep bedroom cool (around 65-68°F)
4. **Caffeine**: Avoid 6+ hours before bedtime

Moderate Impact

5. **Alcohol**: Limit evening consumption (causes fragmentation)
6. **Heavy meals**: Avoid 3+ hours before bed
7. **Bedding**: Comfortable mattress and pillows
8. **Nicotine**: Avoid, especially near bedtime

Lower Impact

9. **Pre-sleep routine**: 30-60 minute wind-down period
10. **Bedroom use**: Reserve for sleep and intimacy only
11. **Screen time**: Avoid 1-2 hours before bed
12. **Activity patterns**: Be active during daylight hours

PHYSICAL RECOVERY

Target: Proper body rest

High Impact

1. **Consistency**: Same sleep/wake times daily (optimizes GH release timing)
2. **Temperature**: Keep bedroom cool (around 65-68°F) - supports deep sleep
3. **Alcohol**: Limit evening consumption (suppresses GH and deep sleep)
4. **Light**: Minimize light exposure during sleep (preserves deep sleep)

Moderate Impact

5. **Heavy meals**: Avoid 3+ hours before bed (affects GH and metabolism)
6. **Activity patterns**: Be active during daylight hours (improves deep sleep)
7. **Noise**: Use white noise or ensure quiet environment
8. **Evening light**: Dim lights 2 hours before bed

Lower Impact

9. **Caffeine**: Avoid 6+ hours before bedtime
10. **Pre-sleep routine**: 30-60 minute wind-down period
11. **Nicotine**: Avoid, especially near bedtime
12. **Screen time**: Avoid 1-2 hours before bed

MENTAL RECOVERY

Target: Proper mental rest

High Impact

1. **Pre-sleep routine**: 30-60 minute wind-down period (reduces worry/rumination)
2. **Consistency**: Same sleep/wake times daily (stabilizes REM patterns)
3. **Alcohol**: Limit evening consumption (severely disrupts REM sleep)
4. **Screen time**: Avoid 1-2 hours before bed (reduces mental stimulation)

Moderate Impact

5. **Caffeine**: Avoid 6+ hours before bedtime (affects sleep quality)
6. **Temperature**: Keep bedroom cool (around 65-68°F)
7. **Bedroom use**: Reserve for sleep and intimacy only (reduces anxiety)
8. **Evening light**: Dim lights 2 hours before bed

Lower Impact

9. **Morning light**: Get bright light exposure upon waking (mood regulation)
10. **Noise**: Use white noise or ensure quiet environment
11. **Light**: Minimize light exposure during sleep
12. **Heavy meals**: Avoid 3+ hours before bed

Sources:

1. Alanazi, A. M. M., et al. (2023) Sleep hygiene practices and its impact on mental health and functional performance among adults in Tabuk City: A cross-sectional study. Cureus, 15(3), e36221. https://pmc.ncbi.nlm.nih.gov/articles/PMC10105495/

2. Baranwal, N.; et al. (2023) Sleep physiology, pathophysiology, and sleep hygiene, Progress in Cardiovascular Diseases, Volume 77, March–April 2023, Pages 59-69, https://doi.org/10.1016/j.pcad.2023.02.005

3. Cleveland Clinic. (2024) Circadian rhythm: What it is, how it works & what affects it. https://my.clevelandclinic.org/health/articles/circadian-rhythm

4. De Pasquale, C., et al. (2024) Sleep hygiene – What do we mean? A bibliographic review. Sleep Medicine Reviews, 75, 101930. https://www.sciencedirect.com/science/article/pii/S1087079224000340

5. Hirohama, K., et al. (2024) The effects of nonpharmacological sleep hygiene on sleep quality in nonelderly individuals: A systematic review and network meta-analysis of randomized controlled trials. PLOS One, 19(6), e0301616. https://journals.plos.org/plosone/article?id=10.1371/journal.pone.0301616

6. Kholghi, M., et al. (2023) Re-considering the role of sleep hygiene behaviours in sleep: Associations between sleep hygiene, perceptions and sleep. International Journal of Behavioral Medicine, 30(5), 651-661. https://link.springer.com/article/10.1007/s12529-023-10212-y

7. National Heart, Lung, and Blood Institute. (n.d.). How sleep works - Your sleep/wake cycle. https://www.nhlbi.nih.gov/health/sleep/sleep-wake-cycle

8. Redeker, N. S. (2023) Physiology, pathophysiology, and sleep hygiene. PubMed. https://pubmed.ncbi.nlm.nih.gov/36841492/

9. Redline, S., Berger, N. A., & Watson, N. F. (2024) Exploring the role of circadian rhythms in sleep and recovery: A review article. Frontiers in Neuroscience, 18, 1221196. https://pmc.ncbi.nlm.nih.gov/articles/PMC11221196/

10. Sleep Foundation (2020) What is circadian rhythm? https://www.sleepfoundation.org/circadian-rhythm

11. StatPearls (2023) Physiology, circadian rhythm. https://www.ncbi.nlm.nih.gov/books/NBK519507/
