# Supplementary material for: Personalized Machine Learning Intervention to Improve Sleep Quality Using Wearable Technology in Healthy Middle-Aged Adults From Mexico City: Protocol for a Pilot Randomized Controlled Trial
Source: JMIR Res Protoc. 2026 Jan 6;15:e76415. doi: 10.2196/76415 (PMC12773695; doi:10.2196/76415)
Supplement: Multimedia Appendix 2 [file resprot-v15-e76415-s002.pdf]

Power analysis table showing power for different effect sizes

ANCOVA power calculations for n=16 per group (α=0.05, one-tailed):

Standardized effect size (Cohen's d)	Statistical Power
0.30 (small-moderate)	0.12
0.50 (moderate)	0.26
0.80 (large)	0.58
1.00 (very large)	0.87

Interpretation: This sample size provides adequate power (>80%) only for very large effects (d≥1.0). For moderate effects typical in sleep research (d=0.3-0.7), power is insufficient, consistent with this study's role as a feasibility-focused pilot trial.
