# Supplementary material for: Personalized Machine Learning Intervention to Improve Sleep Quality Using Wearable Technology in Healthy Middle-Aged Adults From Mexico City: Protocol for a Pilot Randomized Controlled Trial
Source: JMIR Res Protoc. 2026 Jan 6;15:e76415. doi: 10.2196/76415 (PMC12773695; doi:10.2196/76415)
Supplement: Multimedia Appendix 3 [file resprot-v15-e76415-s003.pdf]

CONTRATO DE CONFIDENCIALIDAD Y USO DE DATOS

I. LAS PARTES

Cláusula 1: Identificación de las Partes con identidad y domicilio del responsable que recaba los datos personales.

Para los fines del presente contrato, las partes se identifican de la siguiente manera:

1.1. ________________________________, con domicilio _____________________

_________________, teléfono ___________ y correo electrónico ______________ en su calidad de Investigador, en adelante denominado "el Investigador".

1.2. _______________________________, con domicilio en ___________________

____, en su calidad de Participante, en adelante denominada "el Participante".

1.3 Ambas partes, en adelante conjuntamente denominadas "las Partes", acuerdan celebrar el presente contrato en fecha ___________________________________ bajo los términos y condiciones que se detallan a continuación.

II. OBJETO DEL CONTRATO

Cláusula 2: Del objeto del contrato con las finalidades del tratamiento de datos considerando:

2.1. El Investigador está llevando a cabo una investigación sobre el estudio de proyecto de tesis titulado " Metodología de Aprendizaje Máquina para Mejorar la Calidad del Sueño usando Tecnologías Portátiles (Machine Learning Approach to Enhance Sleep Quality using Wearable Technologies)" (en adelante, la "Investigación").

2.2. Que el Participante desea participar en la Investigación y, en ese contexto, proveerá y tendrá acceso a información confidencial relacionada con la Investigación.

2.3. Que el Investigador y el Participante desean proteger la confidencialidad de dicha información y que el presente alcance incluye el aviso de privacidad.

Se acuerda lo siguiente:

III. DEFINICIONES

Cláusula 3: De las definiciones:

3.1 "Datos Confidenciales" se refiere a toda información y datos proporcionados por el Participante, ya sean escritos, electrónicos o verbales, que se relacionen con el estudio de tesis y que no sean de conocimiento público.

3.2 "Anonimato" se refiere a el procedimiento mediante el cual los Datos Personales o los Datos Personales Sensibles no pueden asociarse al titular ni permitir, por su estructura, contenido o grado de desagregación, la identificación del mismo, también conocido como "Disociación".

3.3 "Datos Personales" se refiere a cualquier información concerniente a una persona física identificada o identificable.

3.4 "Datos Personales Sensibles" se refiere a aquellos datos personales que afecten a la esfera más íntima de su titular, o cuya utilización indebida pueda dar origen a discriminación o conlleve un riesgo grave para éste. En particular, se consideran sensibles aquellos que puedan revelar aspectos como origen racial o étnico, estado de salud presente y futuro, información genética, creencias religiosas, filosóficas y morales, afiliación sindical, opiniones políticas, preferencia sexual. Expresamente para esta investigación de lo que se trata de este tipo de datos son los relacionados con los datos biomédicos a través de los sensores del reloj inteligente proporcionado y los datos declarados por el Participante de forma subjetiva en el llenado del cuestionario del Pittsburg Sleep Quality Index que se refiere por sus siglas en inglés al cuestionario PSQI siendo todos relacionados para el estudio del sueño que es el objeto de esta investigación.

3.5 "Reloj Inteligente" se refiere al dispositivo grado comercial de la marca Samsung, modelo Galaxy 4 de 40 mm de diámetro de carátula y un peso aproximado de 30.3 gramos para la recolección de los datos utilizará algunos o todos los siguientes sensores: acelerómetro, barómetro, giroscopio, sensor geomagnético, sensor de luz, sensor óptico de frecuencia cardíaca, sensor cardíaco eléctrico, sensor de análisis de impedancia bioeléctrica.

IV. OBLIGACIONES DEL INVESTIGADOR

Cláusula 4: De las obligaciones del Investigador incluyendo las opciones y medios que el responsable ofrezca a los titulares para limitar el uso o divulgación de los datos:

4.1 Confidencialidad: El Investigador se compromete a mantener la confidencialidad de los Datos Confidenciales recibidos del Participante y no revelarlos a terceros sin el consentimiento previo y por escrito del Participante.

4.2 Uso de Datos Restringido con Anonimato: Los Datos Confidenciales serán utilizados en modo Anonimato y exclusivamente para el estudio de tesis y para fines académicos relacionados con el proyecto. No se utilizarán para fines comerciales ni se divulgarán fuera del contexto del estudio. Los datos relacionados directamente con el Participante solo le serán comunicados al Participante.

4.3 Seguridad y Medidas de Protección: El Investigador tomará las medidas necesarias para proteger los Datos Confidenciales usando el modo Anonimato protegiendo así también de accesos no autorizados y de cualquier forma de pérdida, alteración o divulgación indebida.

4.4. Proporcionar los Dispositivos Electrónicos: Para el estudio el Investigador prestará un reloj inteligente marca Samsung modelo Galaxy 4 y un celular sin chip emparejado al reloj inteligente.

V. OBLIGACIONES DEL PARTICIPANTE

Cláusula 5: De las obligaciones del Participante:

5.1 Precisión de Datos: El Participante se compromete a proporcionar datos precisos y completos al Investigador para el desarrollo del estudio.

5.2 Uso y cuidado de los Dispositivos Electrónicos: Para el estudio el participante recibirá en calidad de préstamo para el estudio un reloj inteligente marca Samsung modelo Galaxy 4 y un celular sin chip emparejado al reloj inteligente y el Participante se compromete a su debido uso y cuidado. El uso incluye recargar la batería de ambos equipos para que sean emparejados cada 24 horas el reloj inteligente con el celular. Así también el Participante debe usar el reloj inteligente como mínimo durante el tiempo para el sueño y si así lo desea la mayor cantidad de tiempo posible colocando el reloj inteligente en su muñeca con batería y encendido para que los sensores puedan recolectar los datos. Ambos deben ser devueltos a la terminación del estudio o de ser suspendido antes de su terminación por cualquiera de las partes del presente contrato.

5.3 Consentimiento: El Participante concede el consentimiento para que sus datos sean utilizados conforme a lo establecido en el presente contrato, entendiendo que su participación es voluntaria y puede ser retirada en cualquier momento mediante notificación escrita al correo a00450388@tec.mx para realizar la devolución del equipo prestad para esta investigación.

VI. EXCEPCIONES A LA CONFIDENCIALIDAD

Cláusula 6: La obligación de confidencialidad no se aplicará a la Información Confidencial que:

a. Sea o se convierta en información de dominio público sin violación de este contrato por parte del Participante.

b. Sea conocida por el Participante antes de su divulgación por parte del Investigador y no esté sujeta a una obligación de confidencialidad.

c. Sea divulgada por el Participante en cumplimiento de una orden judicial o de una autoridad competente, siempre y cuando el Participante notifique al Investigador con suficiente antelación para permitir que el Investigador tome medidas para proteger la Información Confidencial.

VII. DURACIÓN DEL COMPROMISO

Mientras que la duración del estudio se estima en 60 días naturales, el compromiso de confidencialidad y el uso de los Datos Confidenciales permanecerán en vigor durante la duración del estudio y por un período de 1 año después de su finalización, a menos que se acuerde lo contrario por ambas partes por escrito.

VIII. LIMITACIÓN, DEVOLUCIÓN O DESTRUCCIÓN DE DATOS

Esta cláusula incluye los medios para ejercer los derechos de acceso, rectificación, cancelación u oposición, de conformidad con lo dispuesto en la Ley Federal de Protección de Datos Personales en Posesión de los Particulares. El Participante podrá en cualquier momento hacer llegar al Investigador al correo electrónico establecido en la cláusula 1.1 a00450388@tec.mx solicitud para para limitar el uso o divulgación de los datos o la devolución o la destrucción de los datos. Y al concluir el estudio o a solicitud del Participante, el Investigador deberá devolver o destruir todos los datos y documentos que contengan Datos Confidenciales con la única excepción del presente contrato que es prueba fehaciente del acuerdo entre las partes, asegurando que no queden copias en posesión o control del Investigador.

IX. TRANSFERENCIAS DE DATOS

Por la naturaleza de la investigación no se realizará ninguna transferencia de datos por lo que no se establece un procedimiento y medio por el cual el Investigador comunicará a los titulares de cambios al aviso de privacidad, de conformidad con lo previsto en la Ley Federal de Protección de Datos Personales en Posesión de los Particulares.

X. CONSENTIMIENTO INFORMADO

Las partes firman este contrato prueba de conformidad de todos los términos y condiciones descritos en el presente contrato manifestando que no existe dolo, error o mala fen en la celebración del presente contrato y para el caso de conflicto en su interpretación o su cumplimiento se sujetan a la jurisdicción de los tribunales locales del Estado de México con residencia en Atizapán, renunciando a cualquier otro fuero que pudiera corresponderles en razón del domicilio presente, futuro o por cualquier otra causa que originara una competencia distinta con la fecha estipulada en la cláusula 1.3.

Firma: ____________________	*Firma:* ____________________
Nombre: ____________________	*Nombre:* ____________________
El Investigador	*El Participante*
