# Supplementary material for: Personalized Machine Learning Intervention to Improve Sleep Quality Using Wearable Technology in Healthy Middle-Aged Adults From Mexico City: Protocol for a Pilot Randomized Controlled Trial
Source: JMIR Res Protoc. 2026 Jan 6;15:e76415. doi: 10.2196/76415 (PMC12773695; doi:10.2196/76415)
Supplement: Multimedia Appendix 4 [file resprot-v15-e76415-s004.pdf]

CARTA COMPROMISO

I. LAS PARTES

Identificación de las Partes:

1.1. ________________________________, con domicilio __________________

_________________, teléfono ___________ y correo electrónico ______________ en su calidad de Investigador, en adelante denominado "el Investigador".

1.2. _______________________________, con domicilio en ____________________

____,

y correo electrónico ____ en su calidad de Participante, en adelante denominada "el Participante".

1.3 Ambas partes, en adelante conjuntamente denominadas "las Partes", acuerdan celebrar el presente contrato en fecha ___________________________________ bajo los términos y condiciones que se detallan a continuación.

II. CONDICIONES DEL PRÉSTAMO

Cláusula 2: Como complemento al Contrato de CONTRATO DE CONFIDENCIALIDAD Y USO DE DATOS se formaliza el compromiso respecto al préstamo del equipo de tecnología que a continuación se detalla:

Descripción del Equipo:

Tipo de Equipo: Reloj Inteligente

- *Cantidad: 1*
- *Marca y Modelo: Samsung Galaxy 4*
- *Número de Serie: ____*

Tipo de Equipo: Celular

• Cantidad: 1

• Marca y Modelo: Samsung ______________________________

• Número de Serie: ____

Condiciones del Préstamo:

1. Uso Adecuado: El Prestatario se compromete a utilizar el equipo exclusivamente para fines educativos para la investigación, manteniéndolo en condiciones adecuadas de funcionamiento y evitando cualquier uso que pueda causarle daño.

2. Cuidado y Mantenimiento: El Prestatario deberá tomar todas las precauciones necesarias para proteger el equipo de posibles daños físicos, virus informáticos, o cualquier otro riesgo que pueda afectar su funcionalidad. En caso de pérdida, daño, o robo durante el cuidado del Participante se compromete a entregar equipo en condiciones similares a las entregadas en préstamo.

3. Devolución del Equipo: El Prestatario devolverá el equipo en el mismo estado en que fue entregado, salvo el desgaste normal por uso. La devolución deberá realizarse en un plazo máximo de 5 días después de finalizado el período de préstamo o cuando así lo requiera el prestador.

4. Firmas: Ambas partes firman esta carta en señal de aceptación y compromiso con las condiciones establecidas.

Agradezco de antemano su cooperación y compromiso para garantizar el adecuado uso y cuidado del equipo.

Firma: ____________________	*Firma: ____________________*
Nombre: ____________________	*Nombre: ____________________*
Carácter: Prestador	*Carácter: Prestatario*
